# Tunable and dynamic soft materials for three-dimensional cell culture

**DOI:** 10.1039/c3sm50217a

**Published:** 2013-03-13

**Authors:** Matthew S. Rehmann, April M. Kloxin

**Affiliations:** a Department of Chemical & Biomolecular Engineering , University of Delaware , Newark , DE 19716 , USA . Email: akloxin@udel.edu; b Department of Materials Science & Engineering , University of Delaware , Newark , DE 19716 , USA

## Abstract

The human body is complex and hierarchically structured, composed of cells residing within the extracellular matrix (ECM) of tissues that are assembled into organs, all working together to complete a given function. One goal of current biomaterials research is to capture some of this complexity outside of the body for understanding the underlying biology of development, repair, and disease and to devise new strategies for regenerative medicine or disease treatment. Polymeric materials have arisen as powerful tools to mimic the native ECM, giving experimenters a way to capture key aspects of the native cellular environment outside of the body. In particular, dynamic materials allow changes in the properties of these ECM mimics during an experiment, affording an additional degree of control for the experimenter. In this *tutorial review*, the basic cellular processes of cell migration, proliferation, and differentiation will be overviewed to motivate design considerations for polymeric ECM mimics, and examples will be given of how classes of dynamic materials are being used to study each cellular process.

## Abstract 2

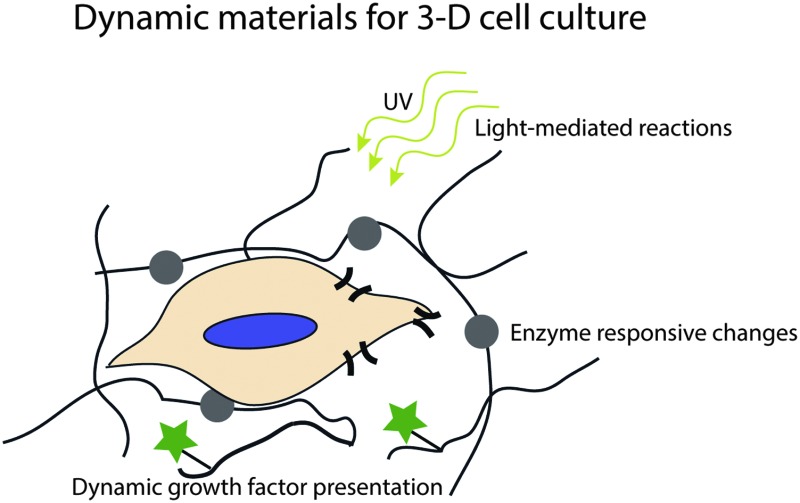
Dynamic biomaterials for cell culture can be used to mimic temporal changes that occur in the native extracellular matrix.

## Introduction

Culturing mammalian cells outside of the body (*in vitro* experiments) is often desirable because of its low cost and simplicity compared to animal models (*in vivo* experiments).^1^ Traditional *in vitro* cell culture involves growing cells on two-dimensional surfaces, which are frequently polystyrene or glass modified with extracellular matrix (ECM) protein(s) or chemical moieties to aid in cell adhesion.^2^ However, the native soft tissue ECM (*E* ∼ 1–200 kPa) is three-dimensional in structure and less rigid than these hard materials (*E* ∼ 3 GPa), and the unnaturally polarized, hard environment presented by traditional tissue culture substrates is very different from what cells experience in the body.^3^ Recent studies indicate that biomaterials may provide a more native-like environment for the cells, causing their behavior *in vitro* to more closely match their behavior *in vivo*.^4^ For example, muscle stem cells expanded on materials that mimic the modulus of muscle retain the capacity to regenerate muscle *in vivo*, whereas these cells expanded on stiffer polystyrene materials lose that regenerative capacity.^5^ Additionally, breast cancer cells cultured in two dimensions on naturally derived ECM protein matrices *in vitro* do not respond the same to chemotherapeutic agents as breast cancer cells *in vivo* or breast cancer cells cultured in three dimensions within these matrices.^6^ While two-dimensional culture on polystyrene and glass remains an important tool, these studies demonstrate the need for soft material-based ECM mimics in the study of cell biology *in vitro*.

**Fig. 1:**
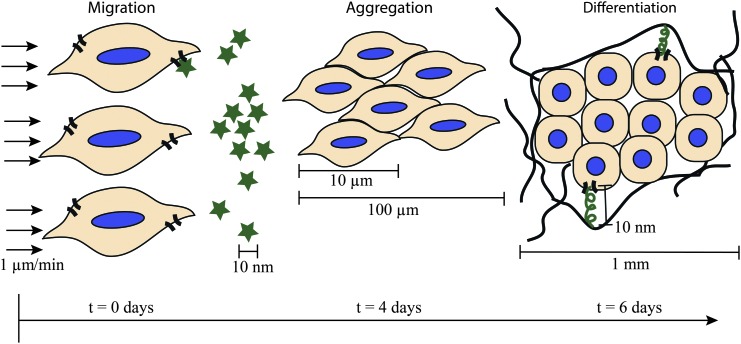
Complex cellular processes key time and size scales. The formation of pre-cartilage condensations illustrates the complex nature of cellular processes and the need for control of the cell microenvironment over multiple time and length scales for examining them. The cell microenvironment is highly dynamic, and cells migrate, aggregate, and begin synthesizing ECM proteins within the first week of embryonic development (chicken wing model).^9^ The cells migrate in response to chemotactic agents (represented by stars) or physical gradients. Once the cells aggregate, they begin differentiating, accompanied by changes in gene expression and protein production. Cells interact with the matrix they synthesize through ligands (squiggles) and cell receptors (curved lines on cell border).

Soft biomaterials can recapitulate many biophysical and biochemical cues of the ECM, such as modulus and integrin-binding moieties, which influence numerous cellular processes.^7^ However, static soft biomaterials do not capture the temporal changes that are characteristic of the native cellular microenvironment, such as ECM protein remodeling and growth factor secretion. Since these temporal changes are key regulators of cell function and fate, materials are needed that afford property control during cell culture. In particular, dynamic extracellular cues regulate the fundamental processes of cell *migration, proliferation, and differentiation*.^8^ A well-studied, illustrative example is cartilage development (Fig. 1). Cartilage development begins with the formation of pre-cartilage condensations,^9^ where cells migrate together, tightly aggregate to form a ‘scaffold’,^10^ and subsequently decrease proliferation.^11^ Cell–cell contacts in these aggregates initiate signaling pathways for differentiation into cartilage cells, chondrocytes, which begin producing collagen II, an important and characteristic ECM protein of cartilage. The maintenance of chondrogenic activity by these cells requires the production of appropriate growth factors as well as dynamic regulation of ECM protein expression. For example, the ECM of cartilage is initially rich in collagen I, aiding in cell proliferation, but the collagen I is eventually replaced by collagen II.^12^ Similarly, the early cartilage ECM is rich in fibronectin, promoting cell adhesion and mediating cell interactions that lead to chondrogenic differentiation, but fibronectin is completely absent from mature cartilage.^12^ These observations have motivated a number of *in vitro* studies to better understand these complex biological processes and mimic them for tissue regeneration.^13,14^

To study dynamic processes like these, model systems are required that not only mimic biochemical and biophysical ECM cues but also give the experimenter control over material properties in time and space.^15^ When designing materials for biological studies, it is important to consider that a typical biological process spans multiple time scales. For example, at a cellular level, cells respond to extracellular signals secreted by neighboring cells, including growth factors, cytokines, and extracellular matrix proteins. If the extracellular signal initiates a change in gene expression, requiring protein synthesis, the cell responds typically within minutes to hours.^16^ If the signal initiates changes that do not require protein synthesis, the cell responds typically within milliseconds to minutes.^16^ At a tissue level, processes such as wound healing involve a cascade of events that take place over days to months; for example, skin would healing involves inflammation, blood clot formation, migration and proliferation of skin cells, and ECM synthesis.^17^ At a whole organism level, human development and maturation from embryogenesis to adulthood takes months to years.^18^

Soft materials have been developed that enable property control over each of these time scales. Reactions involving photochemistry, including photoaddition^19^ and photodegradation,^20^ enable material property manipulation over the course of seconds to minutes. Rates of biomaterial response to enzymatic and hydrolytic reactions span a large range (from minutes to years) and can be tuned by varying the biomaterial composition; however, it is common to see enzymatic reactions that affect biomaterial properties on a time scale of hours to days, and hydrolytic reactions that affect properties on a time scale of days to years.^21^ Reaction selection in material design thus is dictated by property changes that occur on time scales commensurate with cellular responses.

Cellular processes not only change over time but also vary in space, and mimicking the *in vivo* cellular microenvironment requires consideration of these different size scales. A typical mammalian cell has a diameter in the range of 10–100 μm.^22^ However, many biological processes, such as bone morphogenesis, occur on the millimeter or centimeter length scales,^23^ and ligands, such as peptides and proteins, affect cell behavior through receptor-binding on the nanometer size scale.^24^

Like time scales, consideration of size scales is important when designing a biomaterial. On the millimeter and centimeter length scale, the overall geometry of a biomaterial can be controlled by forming it in a mold of the desired shape and size.^25^ On the micrometer length scale, material characteristics, such as surface topography and biomolecule concentration gradients, can be controlled by techniques such as soft lithography,^26^ photolithography,^27^ or microfluidics.^28^ It can be challenging to manipulate biomaterial characteristics on the nanometer length scale, but techniques such as those that utilize self assembly or nanocontact printing are being employed to control initial material properties on this size scale.^29^

In this *tutorial review*, we overview the critical biological events that occur during cell migration, proliferation, or differentiation, with a focus on relevant time and size scales over which these events occur. We discuss soft material-based approaches to study each of these cellular processes within controlled dynamic three-dimensional (3D) culture environments. In the context of this review, a dynamic material is one that has chemical or physical attributes that change over the course of an experiment, and a tunable material is one that has chemical or physical attributes that can be manipulated by the researcher. The primary focus of the review is on hydrogels, although other biomaterials, such as electrospun fibrous scaffolds, also are available for cell culture.^30^ Hydrogels are highly hydrated, crosslinked polymer networks that can be formed from a wide variety of natural and synthetic materials, affording a large degree of tunability in their chemical and physical properties in both space and time. Hydrogel-based materials thus serve well as mimics of the dynamic native ECM of many soft tissues.^31^

Each section of this *tutorial review* overviews a specific cellular process and presents examples of insights gained from *in vitro* cell culture studies with biomaterials. The sections are not intended to be exhaustive or exclusive, as there is often a significant amount of overlap between materials to study different biological processes. For example, growth factors, such as basic fibroblast growth factor, frequently mediate cell migration, cell proliferation, and cell differentiation,^32^ but they are discussed here in the context of cell proliferation. This *tutorial review* is intended to be an introduction to the use of dynamic materials for cell culture applications, with illustrative examples to improve the reader's understanding.

## Cell migration

### Critical spatiotemporal signals involved in cell migration

Cell migration, or the movement of cells to specific locations, is a central phenomenon in cellular biology. Cell migration plays a role in the development of diseases and medical conditions, including osteoporosis, rheumatoid arthritis, and cancer, and in the initiation of tissue morphogenesis during embryonic development.^33^ To design materials to understand cell migration, we must examine relevant biochemical events involved in migration and consider their size and time scales.

Typically, migration first involves cell polarization in response to signals from the environment, such as chemoattractants, ECM proteins, or growth factors.^34^ This causes the cells to form ‘front’ and ‘back’ ends, giving directionality to the cell movement. Polarization is followed by the extension of protrusions, which are stabilized by adhesion to ECM proteins or nearby cells.^33^ The cell then moves forward, and the protrusions are disassembled in the cell rear.^33^ Cells have a bimodal response to ligand concentration, where maximum migration speeds and distances are typically observed for intermediate ligand concentrations rather than low or high ligand concentrations.^35^

Single cells can migrate by two general mechanisms: amoeboid migration and mesenchymal migration.^36^ Amoeboid migration involves motion by rounded or ellipsoidal cells undergoing rapid expansion and contraction; it requires weak interactions with nearby ECM proteins and allows the cells to maneuver around ECM barriers.^36^ A typical rate for amoeboid migration is 2–30 μm min^–1^.^36^ In contrast, mesenchymal migration involves spindle-shaped elongation and typically requires the use of proteases, such as matrix metalloproteinases (MMPs), to digest the nearby ECM proteins and allow the cells to move. A typical rate for mesenchymal migration is an order of magnitude slower than amoeboid migration (about 0.5–2 μm min^–1^).^36^

### Dynamic materials for probing cell migration: enzyme-responsive materials

**Fig. 2:**
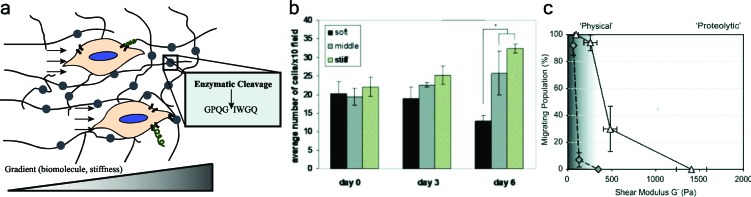
Enzyme responsive materials for examining cell migration. (a) Cells migrate through enzymatically degradable materials (enzymatically degradable units depicted by circles) in response to chemical or physical gradients. Cells migrate along gradients, such as from regions of low chemotactic agent concentration to high chemotactic agent concentration, cleaving proteolytically degradable units, such as GPQGIWGQ. (b) Hadjipanayi *et al.* studied the migration of human dermal fibroblasts in response to a gradient of matrix stiffness in a collagen hydrogel. Cells preferentially migrated towards the stiff region of the material. In their study, the stiff region also had a higher density of integrin-binding ligands. Reprinted from ref. 37 with permission from John Wiley and Sons. Copyright (2009). (c) Ehrbar *et al.* studied the migration of mouse preosteoblastic cells in synthetic enzymatically degradable PEG hydrogels. At low moduli, cells can migrate in degradable (white triangles) and non-degradable hydrogels (grey diamonds). At higher moduli, proteolysis and matrix degradability are required for migration, and migration decreases as modulus increases. Reprinted from ref. 46 with permission from Elsevier. Copyright (2011).

Enzyme-responsive materials provide an *in vitro* model system for studying migration, especially proteolytically mediated mesenchymal migration (Fig. 2a). Many natural biomaterials, such as collagen or fibrin hydrogels, are enzymatically degradable, enabling cells to degrade and remodel their matrix as they respond to stimuli. Derived from animal sources, these natural materials inherently present cells with surroundings that are similar in composition and structure to the *in vivo* cell microenvironment. In particular, since collagen is the most abundant protein in the human body, collagen hydrogels are commonly used as a model system to study cellular processes, including cell migration, *in vitro*. For example, Hadjipanayi *et al.* used collagen hydrogels to show that human dermal fibroblasts preferentially migrate towards stiff regions of a biomaterial in 3D culture (Fig. 2b).^37^ Collagen hydrogels are dynamic, since they can be invaded and remodeled by cells;^38^ tunable, since collagen hydrogel properties can be manipulated by the presence of glutaraldehyde and different gel formation conditions;^39^ and suitable for three-dimensional cell culture.^4^

While natural materials present many biochemical and biophysical cues to cells, synthetic materials are typically more biologically inert, more amenable to manipulation and customization, and subject to less batch-to-batch variation, providing blank slates for the presentation of select ECM cues.^40^ Some synthetic materials, such as poly(ethylene glycol) (PEG) hydrogels, have been engineered to be enzymatically degradable and serve as controlled ECM mimics for studying migration. For example, Raeber *et al.* co-polymerized a protease degradable dicysteine peptide crosslinker (GPQGIWGQ) with a multifunctional PEG vinyl sulfone monomer to create an enzymatically degradable hydrogel for studying cell migration.^41^ This collagen-derived enzymatically degradable peptide is susceptible to cleavage by a multiple matrix metalloproteinases (MMPs), including MMP-1, -2, -3, -7, -8, and -9.^42,43^ The authors observed migration speeds of approximately 0.2 μm min^–1^ in the PEG gels, significantly slower than migration speeds of 0.55 μm min^–1^ observed in their collagen gels, which had larger pore sizes.

Building upon the work by Raeber *et al.*,^41^ Schwartz *et al.* used a multifunctional PEG norbornene monomer to react with a protease degradable dicysteine peptide crosslinker *via* photoinitiated free radical polymerization.^44^ The photoinitiated step growth mechanism enables facile light-triggered hydrogel formation, and while not utilized by Schwartz *et al.*, biochemical signals can be added at later timepoints by incorporating excess norbornene during gel formation.^45^ Schwartz *et al.* encapsulated human HT-1080 fibrosarcoma cells in the material and observed their migration in response to varying concentrations of the integrin-binding adhesive peptide sequence RGDS (arginine–glycine–aspartic acid–serine, presented at 0 to 1.5 mM). The authors demonstrated that the HT-1080 cells can simultaneously use aspects of both amoeboid and mesenchymal migration: the cells retained significant amoeboid character even though they were migrating by proteolytic mechanisms.

Ehrbar *et al.* developed a PEG hydrogel that was both formed and degraded by enzymatic reactions for the study of cell migration.^46^ The authors covalently bound a peptide mimicking the Factor XIII crosslinking site (FKGG) to multifunctional PEG, creating reactive monomers, and added activated Factor XIII to the solution, forming crosslinks between the peptides. They also incorporated a protease-degradable site within the peptide (GPQGIWGQ), allowing the hydrogel to enzymatically degrade. Thus, Ehrbar *et al.* were able to use enzymes to form bioresponsive hydrogels without the addition of chemical initiators, which could be advantageous for cell types that are sensitive to other polymerization conditions. The authors showed that fewer cells migrate when hydrogel stiffness increases (Fig. 2c), and they observed migration speeds on the order of 0.2 to 1.0 μm min^–1^ in these gels.

An advantage of these synthetic approaches over the use of natural biomaterials is that the PEG hydrogels have much smaller pore sizes than the collagen hydrogels, affording controlled studies of proteolytically mediated migration. Typical PEG hydrogels have pore sizes on the order of 10 nm, significantly smaller than the size of a cell, whereas collagen hydrogels have pore sizes on the order of 1–10 μm, which is only slightly smaller than a typical mammalian cell.^41^ Thus, proteolysis is required for migration through these synthetic gels, and they are appropriate for the study of migration when only proteolytic migration modes are desired (*e.g.*, mesenchymal migration). Notably, however, Ehrbar *et al.* observed that cells can migrate by non-proteolytic modes when these gels are formed at low monomer concentrations, perhaps owing to defects in gel formation.^46^ If only proteolytic migration is desired, tests should be run to confirm that cells are not migrating in a protease-independent manner (for example, using protease inhibitors).

In addition to being dynamic, enzymatically degradable PEG hydrogels are tunable, where matrix degradation rates can be varied by changing the identity of the enzyme-responsive unit^47^ or by controlling the initial matrix properties by varying functional group stoichiometry^46^ and monomer molecular weight.^48^ Furthermore, the utility of enzyme-responsive materials is not limited to migration studies.^49^ Todd *et al.* tethered the peptide Fmoc-FRGD to a PEG-acrylate surface; the bulky fluorenylmethoxycarbonyl (Fmoc) group sterically prevented interaction between cells and the bioactive RGD ligand.^50^ Upon the application of chymotrypsin, the peptide was cleaved at the phenylalanine (F), removing the Fmoc and exposing the RGD ligand to the cells. In this way, RGD ligand density was dynamically tuned during culture by applying chymotrypsin at time points of interest. This approach could be used for dynamic user- or cell-directed biomolecule presentation in either two- or three-dimensional culture.^51^

Thus, enzyme-responsive materials allow for the cells to manipulate their own microenvironment as they respond to stimuli. The materials can be engineered by changing the identity of the enzyme-responsive unit. Enzyme-responsive materials are appropriate for a variety of studies, including studies of cell migration, where proteolytic activity is often a major regulatory factor.

## Cell proliferation

### Critical spatiotemporal signals involved in cell proliferation

**Fig. 3:**
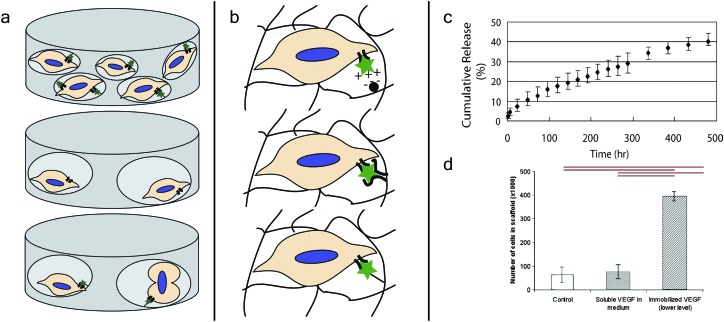
Growth factor presenting dynamic materials for controlling cell proliferation. (a) Cells need nutrients, mitogens, and space to proliferate, providing design criteria for biomaterials to direct and study this process. For example, cells can receive signals from mitogens (stars) to promote proliferation, but without the space for additional cells, they will not proliferate (top). In contrast, while cells may have space to proliferate, they will not proliferate without appropriate mitogen signaling (middle). Cells given both mitogens and space proliferate (bottom). (b) There are several methods by which growth factors can be dynamically presented by biomaterials towards regulating cellular processes such as proliferation. Growth factors can be electrostatically attracted to a moiety incorporated within the material (top), sequestered by non-covalent interactions with an affinity molecule (middle), or covalently immobilized within the biomaterial through a degradable linker (bottom). (c) Tae *et al.* released vascular endothelial growth factor from hydrogels with heparin, controlled *via* electrostatic interactions. Approximately 40% of the growth factor was released over the course of about three weeks. Reprinted from ref. 63 with permission from Taylor & Francis. Copyright (2006). (d) Shen *et al.* measured proliferation of endothelial cells in collagen hydrogels in response to vascular endothelial growth factor. The authors saw significantly greater proliferation when the growth factor was immobilized in the hydrogel compared to control hydrogels without the growth factor or hydrogels where the growth factor was presented in soluble form. Reprinted from ref. 67 with permission from Elsevier. Copyright (2008).

Cell proliferation is the process of cell division, leading to an increase in cell number. For a cell to proliferate, the nutrients available in the surrounding environment must be sufficient. Furthermore, the appropriate mitogens must be present in order for proliferation to take place (Fig. 3a). Mitogens are extracellular molecules, typically peptides or proteins, that regulate cell proliferation by initiating intracellular signaling pathways associated with mitosis.^52^ Although the terms cell proliferation and cell growth are often used interchangeably, cell growth is an increase in cell mass through the synthesis of macromolecules and is stimulated by growth factors.^52^ Cell growth and cell proliferation are necessarily coordinated, and many proteins, such as platelet derived growth factor and epidermal growth factor, can act as both mitogens and growth factors.

Cells also need space to grow and divide for proliferation to occur (Fig. 3a). This is an especially important consideration for biomaterial design in 3D culture, since the pore size of many synthetic materials is smaller than the size of a cell.^41^ If proliferation is desired in a non-degradable material, the pores must be sufficiently large to accommodate an additional 10–100 μm cell.^22^ If cells are grown in an enzymatically degradable material, the cells can degrade the surrounding matrix to create pores for proliferation.^53^ If the cells are not grown in an enzymatically degradable material, they can be given space by hydrolytic, photolytic, or other degradation mechanisms.^54^ When proliferation is desired, consideration must be given to the rates of degradation and size of pore formation towards matching the time scale of proliferation.

To proliferate, most mammalian cells go through a four-phase cycle called the mitotic cell cycle. The cycle proceeds from G_1_ phase to S phase to G_2_ phase to M phase and then repeats. The gap phases, G_1_ and G_2_ phase, have proliferation checkpoints to ensure replication proceeds without errors. Cells can remain in G_1_ for long periods of time if external conditions are unfavorable for replication.^52^ In S phase, the cell replicates its DNA. In M phase, the cell goes through nuclear division (mitosis) and cytoplasmic division (cytokinesis), turning one cell into two. A typical time scale for progression through the cell cycle for mammalian cells is 12 hours to a few days.

If extracellular conditions are unfavorable for proliferation, the cell also can exit the cell cycle and enter a resting state known as quiescence. Quiescent cells exhibit low metabolism and protein synthesis, and quiescence is typically reversible, where cells can re-enter the cell cycle after being presented with the appropriate stimulatory signals.^55^ This is in contrast to senescence, where cells have permanently lost their ability to proliferate. Senescence *in vitro* is a naturally occurring process that happens after non-cancerous cells undergo a certain number of proliferation events; the exact role of senescence *in vivo* is not well understood, but it has been associated with cancer prevention and natural aging.^56^ Since extracellular signals regulate cell cycle progression, dynamic biomaterials can be designed to probe and direct cell fate.^57^

### Dynamic materials for probing cell proliferation: growth factor presenting biomaterials

Growth factors, such as platelet-derived growth factor, epidermal growth factor, and transforming growth factor-β, are of interest partially because of their tendency to promote cell growth and proliferation, but they also regulate other biological processes, such as migration and differentiation.^58,59^ Growth factors can be presented to cells *in vitro* in soluble media; however, this may not be the most biologically relevant method of presentation, since *in vivo* growth factors are typically associated with or sequestered by the ECM.^60^ Researchers have used several strategies to sequester and locally release growth factors from and within biomaterials (Fig. 3b).^61^ Although these materials have primarily been evaluated for controlled drug release, many of them are compatible with 3D cell culture and could be used to release growth factors for cell culture applications.^62^

The most common type of growth factor–ECM interaction *in vivo* is the association of a growth factor with heparin or heparan sulfate.^60^ To mimic this interaction, heparin has been incorporated into a number of biomaterials for the controlled release of growth factors. These growth factor releasing materials are dynamic, since the growth factor is initially associated with the material and then temporally released. For example, Tae *et al.* incorporated heparin into a PEG hydrogel and demonstrated the controlled release of biologically active vascular endothelial growth factor over the course of three weeks (Fig. 3c).^63^ Heparin has the strongest negative charge density of any known biomolecule, and many proteins interact with it electrostatically. To mimic this electrostatic interaction, Freeman *et al.* added sulfate groups to alginate and hyaluronic acid, two types of polysaccharides often used to make hydrogels for cell culture applications.^64^ These materials showed strong electrostatic interactions with a variety of proliferation-inducing growth factors, such as platelet derived growth factor, basic fibroblast growth factor, and hepatocyte growth factor. Furthermore, basic fibroblast growth factor was released from the biomaterial over the course of several days.

An alternative approach to the use of heparin is to incorporate a molecule, such as a peptide, that has high non-covalent binding affinity for a specific protein of interest. McCall *et al.* incorporated peptide sequences that bind non-covalently with high affinity to transforming growth factor-β into PEG hydrogels.^65^ The authors showed that, using this method, bioactive transforming growth factor-β was released over the course of several days. The use of alternatives to heparin, such as affinity peptides, increase the tunability of the materials, since the release rate is dictated by the interactions between the material and the compound being released.^66^

If desired, proteins and other biomolecules can be covalently tethered to biomaterials, rather than non-covalently sequestered. The immobilization of growth factors in biomaterials allows for increased potency, due to the lack of growth factor internalization by cells, and greater persistence of growth factor signaling to cells.^67^ For example, Shen *et al.* immobilized vascular endothelial growth factor in collagen hydrogels and showed that the immobilized growth factor promoted the invasion and proliferation of endothelial cells in those gels more so than an equal concentration of soluble vascular endothelial growth factor.^67^ Immobilization of growth factors also allows for facile methods of forming growth factor gradients in biomaterials, which is useful for directing cell migration, proliferation, and differentiation.^68^ For example, DeLong *et al.* used covalently attached gradients of basic fibroblast growth factor to direct smooth muscle cell behavior.^69^ Although materials with immobilized growth factors are inherently less dynamic than growth factor releasing biomaterials, these growth factor gradients can be added spatiotemporally, for example, using photoaddition techniques,^70^ and materials can be designed to release immobilized growth factors by attachment through degradable linkages.^71^

Growth factor presenting or releasing materials allow for pre-engineered changes in the material composition to occur during culture. The materials can be tuned by changing the affinity of the biomaterial moiety for the released compound of interest and the material crosslink density. Growth factor releasing materials are appropriate for a variety of studies related to dynamic extracellular regulation of cellular functions, including proliferation, migration, and differentiation.

## Cell differentiation

### Critical spatiotemporal signals involved in cell differentiation

**Fig. 4:**
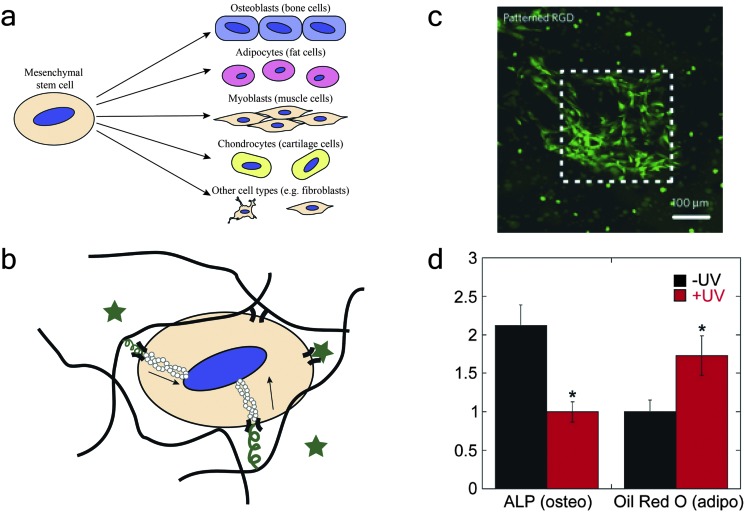
Photoresponsive materials for probing cell differentiation. (a) Stem cells, such as mesenchymal stem cells, show multilineage differentiation potential and can differentiate into a variety of different cell types. (b) Cell differentiation can be regulated by soluble factors (stars), solid-phase integrin-binding peptides (squiggles), and mechanical cues transduced by actin filaments (depicted by hollow circles). (c) DeForest *et al.* used photopolymerization to pattern RGD in specific regions of an enzymatically degradable PEG hydrogel. The authors demonstrate that significant cell spreading is only observed where RGD has been added. Reprinted with permission from Macmillan Publishers Ltd: *Nat. Mater.*, ref. 70. Copyright (2009). (d) Khetan and Burdick used sequential crosslinking to demonstrate the importance of matrix mechanical cues on stem cell differentiation. The authors synthesized a hyaluronic acid hydrogel and cultured cells inside of the gel with (+UV) or without (–UV) additional crosslinking at a later timepoint. The light-mediated addition of crosslinks limited cell spreading (+UV), causing the cells to differentiate down an adipogenic lineage (marked by oil red o). The cells were better able to spread without additional crosslinking (–UV), causing the cells to differentiate down an osteogenic lineage (marked by ALP). Reprinted from ref. 98 with permission from Elsevier. Copyright (2010).

Cell differentiation is a process during which a cell goes from a less specialized to a more specialized cell type. For example, mesenchymal stem cells (MSCs) can differentiate into osteoblasts (bone cells), chondrocytes (cartilage cells), or adipocytes (fat cells) amongst other lineages (Fig. 4a).^72^ During differentiation, gene expression of the cell is altered, leading to changes in protein expression and secretion, and cell differentiation typically takes place on a time scale of days to weeks.^19,20,73,74^ Many recent studies have been focused on the differentiation of stem cells, such as MSCs^75^ or embryonic stem cells (ESCs),^76^ for tissue engineering applications. Additionally, recent interest has arisen for the use of induced pluripotent stem cells (iPSCs) in disease models to understand complex human diseases and towards personalized medicine.^77^

During *in vitro* cell culture, cell differentiation can be induced with outside-in signaling by (1) incorporating soluble factors into the chemical media, (2) incorporating bioactive ligands into the biomaterials, and/or (3) altering the mechanical properties or surface topography of the culture substrate (Fig. 4b).^78^ For example, neuronal differentiation of ESCs can be induced by soluble sonic hedgehog and neurotrophin-3,^79^ and chondrogenic differentiation of MSCs in alginate hydrogels can be enhanced by the presence of the integrin-binding ligand RGDS.^80^ MSC lineage in PEG–silica hydrogels is dictated by gel liquefaction stress,^81^ a measure of matrix stiffness for these three-dimensional gels. Further, MSCs show enhanced osteoblastic differentiation on polymethylmethacrylate substrates with disordered square arrays than on planar polymethylmethacrylate surfaces, demonstrating that substrate topography can regulate cell differentiation.^82^ From these examples, it is clear that biomaterials play a large role in the study of cell differentiation.

Although enzyme-responsive and other dynamic materials have led to progress in the study of cell differentiation, there is a need for user-mediated methods of directly manipulating material behavior. One way this can be achieved is by using photo-responsive biomaterials.

### Dynamic materials for probing cell differentiation: photo-responsive biomaterials

Light-responsive biomaterials typically involve a photoactive group incorporated covalently within the biomaterial, such as a nitrobenzyl ether group,^83^ or a soluble light-responsive unit that interacts/reacts with the biomaterial, such as a photoinitiator.^84^ Photochemistry can achieve rapid material responses with high resolution. Typical size scales for photopatterned features are on the order of 10–100 μm,^20^ the resolution of a typical film-based photomask used to control where irradiation occurs; however, photopatterning resolution on the 1 μm size scale has been reported using two-photon irradiation.^85^ The time scales of photopatterning depend on the speed at which a light-responsive unit reacts, but reactions on the order of seconds to minutes are typical.^20^

When applying photochemistry for cell culture applications, it is critical to consider the cytocompatibility of the system being used. Ultraviolet light, which has been associated with DNA damage,^86^ is typically applied to samples; however, damage can be minimized and cell viability maintained by selecting photolabile groups with absorbances and quantum yields well matched for use with low doses of long wavelength UV or visible light.^87–89^ Furthermore, photoinitiators that generate free radicals, which can damage DNA, proteins, and lipids,^90^ are often used for light-mediated biomaterial formation or modification. Thus, low concentrations of cytocompatible photoinitiatiors, such as Irgacure 2959 (I2959),^91^ lithium phenyl-2,4,6-trimethylbenzoylphosphinate (LAP),^92^ or eosin Y,^93^ should be used for cell studies. Doses of light should be kept low to minimize cell exposure and heating, and appropriate controls should be used to ensure cells are not functioning abnormally in response to exposure to UV light. For example, proliferation should be unaffected, apoptosis should be limited, and there should be no significant activation of p53, a protein which becomes activated upon damage of DNA.^19,94^

Strategies for using light to dynamically tune materials include (1) using light to initiate chemistry, incorporating a moiety into the hydrogel, or (2) using photodegradation to remove a photoactive group after polymerization. For example, bioactive peptide ligands to induce biological behavior can be incorporated into a biomaterial by light-initiated chemistry. Hoffmann and West used photopolymerization to add acrylate-RGDS to PEG-diacrylate hydrogels after polymerization.^95^ DeForest *et al.* extended this approach by using orthogonal chemistries to independently tune initial network structure with cytocompatible azide–alkyne click chemistry and the spatiotemporal incorporation of bioactive ligands with photo-initiated thiol–alkene chemistry (Fig. 4c).^70^ The advantage of these approaches is the ease with which these materials are dynamic and tunable: reactions mediated by photochemistry occur at a location and time dictated by the researcher with light, giving *in situ* spatiotemporal control of the biomaterial properties.

Bioactive peptide ligands can also be removed from a biomaterial by light-mediated chemistry. Kloxin *et al.* synthesized an acrylated photolabile nitrobenzyl ether moiety for incorporation into PEG-diacrylate hydrogels.^20^ The moiety was attached to an RGDS peptide ligand, which was removed 10 days into the cell culture experiment. The authors demonstrated the biological importance of this approach by showing that this dynamic removal leads to an increase in chondrogenesis of MSCs when compared to MSCs that are continually exposed to RGDS. Further, DeForest and Anseth have demonstrated visible light photoaddition and UV light photoremoval of RGDS peptides for adding and removing integrin-binding signals in the presence of cells.^93^

In addition to biochemical cues, recent studies have demonstrated the effect of modulus on cell differentiation. In a seminal study, Engler *et al.* showed that, in the presence of identical media conditions, stem cells differentiated down a lineage that is dictated by the stiffness of the substrate.^96^ Huebsch *et al.* extended this work to 3D cell culture, showing that soft substrates (*E* ∼ 2.5–5 kPa) lead to adipogenic differentiation of MSCs, whereas intermediate substrates (*E* ∼ 11–30 kPa) lead to osteogenic differentiation of MSCs.^97^ Pek *et al.* observed similar results in 3D culture for another mechanical parameter related to matrix stiffness, showing that stem cell lineage is dictated by the liquefaction stress of a PEG–silica gel (the minimum shear stress needed to liquify these thixotropic gels).^81^ Thus, modulus has emerged as an important parameter for determining cell fate, and, as a result, researchers have been working on materials-based approaches to change modulus in time. Khetan and Burdick developed a hyaluronic acid hydrogel that can be stiffened by sequential crosslinking reactions that occur at a user-controlled time.^98^ They showed that increasing the degree of crosslinking in these hydrogels decreased the degree of MSC spreading, increasing adipogenesis of MSCs within these gels (Fig. 4d). Analogously, Kloxin *et al.* used a photodegradable PEG hydrogel to decrease crosslinking at a user-controlled time *via* degradation and observed increased cell spreading with decreased crosslinking density.^20^

Further, surface topography is increasingly recognized as another important physical cue in directing stem cell differentiation,^99^ motivating recent efforts that utilize photochemistry to regulate biomaterial surface topography. For example, Wong *et al.* demonstrated the ability to create positive and negative topographic features in a hydrogel surface over length scales from the nanometer to centimeter range using photodegradation.^100^ Additionally, Kirschner and Anseth used a photodegradable PEG hydrogel to dynamically change topography in the presence of cells, showing that MSCs change their morphology and alignment in response to these changes in temporal subcellular topography.^101^ A complementary approach to dynamically control topography is spatiotemporal wrinkling of PDMS to direct stem cell differentiation.^102^

Thus, photoresponsive materials allow for a user-controlled method for spatiotemporal manipulation of cell microenvironments. Specific changes can be induced in particular regions of the materials at desired times. Photoresponsive materials are appropriate for a variety of studies, including studies of cell differentiation, where precise control of the microenvironment in space and time is often desirable.

## Conclusion

Biomaterials that mimic the dynamic nature of the cell microenvironment enable us to probe and direct cellular processes. Here, we have overviewed soft materials that are discretely enzyme responsive, growth factor presenting, or photoresponsive for examining cell migration, proliferation, or differentiation, all critical processes in tissue regeneration and disease. However, these cellular processes are not decoupled, and likewise, combinations of chemistries reviewed here can be utilized within a single material to investigate complex biological processes (*e.g.*, stem cell homing, proliferation, and differentiation in tissue repair). Further, the tunable and dynamic materials surveyed can be used in conjunction with *in situ* cell monitoring strategies and high throughput assays to examine the bidirectional, temporal interplay between cells and their microenvironment, such as examining fluorescent reporters of gene and protein expression with high throughput image analysis and flow cytometry.^103,104^ In sum, the design of unique soft materials to mimic spatiotemporal biological processes continues to further our understanding of how the microenvironment regulates cell function and fate and pushes the boundaries of materials, biology, and medicine.
